# Effectiveness and Safety of First-Line Pembrolizumab in Older Adults with PD-L1 Positive Non-Small Cell Lung Cancer: A Retrospective Cohort Study of the Alberta Immunotherapy Database

**DOI:** 10.3390/curroncol28050357

**Published:** 2021-10-18

**Authors:** Heidi A. I. Grosjean, Samantha Dolter, Daniel E. Meyers, Philip Q. Ding, Igor Stukalin, Siddhartha Goutam, Shiying Kong, Quincy Chu, Daniel Y. C. Heng, D. Gwyn Bebb, Don G. Morris, Winson Y. Cheung, Aliyah Pabani

**Affiliations:** 1 Department of Oncology, University of Calgary, Calgary, AB T2N4N2, Canada; heidi.grosjean@ucalgary.ca (H.A.I.G.); samantha.dolter@ucalgary.ca (S.D.); daniel.meyers@ucalgary.ca (D.E.M.); istukali@ucalgary.ca (I.S.); Shiying.Kong@albertahealthservices.ca (S.K.); daniel.heng@albertahealthservices.ca (D.Y.C.H.); Gwyn.Bebb@albertahealthservices.ca (D.G.B.); donald.morris@albertahealthservices.ca (D.G.M.); winson.cheung@albertahealthservices.ca (W.Y.C.); 2 Oncology Outcomes, Calgary, AB T2N4N2, Canada; p.ding@ualberta.ca; 3 Faculty of Medicine and Dentistry, University of Alberta, Edmonton, AB T6G2R3, Canada; goutam@ualberta.ca; 4 Cross Cancer Institute, Edmonton, AB T6G1Z2, Canada; quincy.chu@albertahealthservices.ca

**Keywords:** immunotherapy, non-small-cell lung cancer (NSCLC), older adults

## Abstract

The emergence of immunotherapy revolutionized the treatment of non-small-cell-lung cancer (NSCLC), with multiple landmark clinical trials establishing the efficacy of these agents. However, many patients who receive immunotherapy in clinical practice would be considered clinical trial ineligible. One such population that is often under-represented in clinical trials is older adults. In the current study, we evaluated clinical and safety outcomes in this population. Overall, older adults (>70 years of age) and younger adults had comparable clinical outcomes with an equivalent objective response rate (ORR), time to treatment failure (TTF), and median overall survival (*p* = 0.67, *p* = 0.98, and *p* = 0.91, respectively). Furthermore, the safety outcomes were equivalent between the cohorts with similar rates of immune-related adverse events (irAEs), irAE-related hospitalizations, and all-cause hospitalization (*p* = 0.99, *p* = 0.63, and *p* = 0.74, respectively). While older age was not found to impact overall survival, multivariant analysis revealed that a poor Eastern Cooperative Oncology Group (ECOG) status, low body-mass-index (BMI), and poor/intermediate lung immune prognostic index (LIPI) were all associated with worse survival. In conclusion, age does not impact the efficacy or safety of pembrolizumab in NSCLC, and therefore advanced age should not be a deterrent for treating these patients with pembrolizumab. Physicians and care providers can thus focus on other factors that may influence therapeutic outcomes.

## 1. Introduction

In 2018, lung cancer was the leading cause of cancer death worldwide, and represented 11.6% of all new cancer cases globally [1]. The emergence of immunotherapy has revolutionized the treatment of lung cancer and, in particular, non-small-cell-lung cancer (NSCLC), with multiple landmark clinical trials establishing the efficacy of these agents [2,3]. Most recently, the 5-year follow-up data from the KEYNOTE-024 clinical trial showed a median OS (overall survival) of 26.3 months for Programmed Death-Ligand 1 (PD-L1) positive NSCLC patients treated with first-line pembrolizumab, with almost a third of the pembrolizumab group still alive at the 5-year mark [4].

While these clinical trials provide valuable information, the cohorts selected often do not accurately represent patients in a real-world setting. Many trials of pembrolizumab and nivolumab in NSCLC have a median age of >65, and exclude patients with an ECOG PS (Eastern Cooperative Oncology Group Performance Status) above 1, untreated brain metastases, or active autoimmune conditions [5]. However, in clinical practice, many NSCLC patients that would be considered clinical trial ineligible receive immunotherapy [6], and there is limited information on the effectiveness of pembrolizumab in these real-world cohorts.

Older adults carry a large burden of lung cancer rates, with most lung cancer being diagnosed between age 65–74 and a median age at diagnosis of about 70 years old [7]. Despite this, older adults are often underrepresented in lung cancer clinical trials, and even outside of trial settings there are limited data on the use of immunotherapy in older adults [8]. Additionally, there are concerns that older adults may be more vulnerable to treatment-related toxicities, with conflicting data on whether older adults experience more frequent or severe toxicities from immunotherapy [9]. Several studies have examined the efficacy and safety of immunotherapy in older adults with NSCLC [10,11,12], but there remains a shortage of standardized real-world data, as many studies either use multiple IO agents or do not address other potential contributing factors such as prognostication metrics [10,13].

To address this, we conducted a retrospective cohort study specifically examining the age-related effectiveness and safety of first-line pembrolizumab monotherapy in a real-world population of NSCLC patients. Our secondary aim was to examine the baseline clinical and pathological variables that may have prognostic value in the treatment of NSCLC in the era of immunotherapy.

## 2. Materials and Methods

### 2.1. Study Design and Data Collection

We conducted a retrospective cohort study using the Alberta Immunotherapy Database (AID). AID includes consecutive patients ≥ 18 years of age treated with immunotherapy for metastatic melanoma or NSCLC in a non-clinical trial setting at one of two tertiary care centers in Alberta, Canada (Tom Baker Cancer Centre (Calgary, Alberta) or Cross Cancer Institute (Edmonton, Alberta)) between 1 January 2010, and 31 December 2019. The total database encompassed 1378 unique patients. Patients were identified using provincial pharmacy records, and individual charts were reviewed retrospectively in order to complete data collection based on a standardized template.

For the present study, analyses were limited to patients treated with first-line pembrolizumab for NSCLC who had a confirmed PD-L1 tumor proportion score ≥ 50%, which was based on the KEYNOTE-024 trial [3]. The PD-L1 IHC assay used in Alberta, Canada is the Agilent Technologies Inc.^®^ PD-L1 IHC 22C3 pharmDx assay. Baseline clinical, pathological, and laboratory bases data were collected for each patient. If data were not available within 30-days prior to the initiation of therapy, then they were considered unavailable. Chart review and data collection occurred was initiated on 1 July 2017, with 1 October 2020 being the date of data cut-off.

Approval for the study was obtained through the Health Research Ethics Board of Alberta, the Cancer Committee (HREBA.CC-19-0380). Individual patient consent was not required due to the retrospective nature of the study.

### 2.2. Outcomes

The primary outcome of interest was overall survival (OS), which was calculated from the date of first pembrolizumab until death from any cause, or the date of last follow-up. Patients alive at the date of last follow-up were censored. Additional outcomes included time to treatment failure (TTF), which was calculated from the date of first pembrolizumab until the date of therapy cessation for any reason, and the objective response rate (ORR), which was defined as the proportion of patients achieving a complete or partial radiographic response based on Response Evaluation Criteria in Solid Tumors (RECIST) version 1.1. Exploratory outcomes of interest, including the proportion of patients at the 3-, 12-, and 24-month landmarks, and the proportion of patients receiving subsequent therapy (defined as any additional chemotherapy given after cessation of pembrolizumab) were also collected.

The primary safety outcome was the proportion of patients who developed any clinically significant immune-related adverse event (irAE), which was defined as either receiving treatment with systemic corticosteroids or requiring a treatment delay. Only the most clinically significant and severe irAE was recorded for each patient. Secondary safety outcomes included the proportion of patients with irAE requiring hospitalization, as well as the proportion requiring hospitalization for any reason while on pembrolizumab or within one month of completing therapy.

### 2.3. Statistical Analysis

Data were analyzed between 1 April and 30 May 2021. Baseline demographic data were described using proportions (%), and the Pearson’s Chi-squared test was used to evaluate the differences in the baseline characteristics between groups. OS and TTF were calculated using the Kaplan−Meier method, and were compared between age groups using a log-rank test. All other clinical and safety outcomes were compared between age groups using a Pearson’s Chi-squared test and Fisher’s exact test.

A univariate Cox-proportional hazard model was constructed with key clinical characteristics to assess their impact on survival. Characteristics with a cut-off *p* value of <0.2 in the univariate analysis were then included in a multivariate Cox-proportional hazard regression analysis to determine their impact on survival. Age was also included in the multivariate analysis despite not meeting the *p*-value threshold, as it was our variable of interest for this study. Missing data were handled by the case-deletion method. Data were analyzed using R [14].

## 3. Results

### 3.1. Patient Characteristics

In total, 327 patients were identified as receiving first line pembrolizumab for the treatment of NSCLC with a PD-L1 TPS ≥ 50%. Baseline clinical and pathological characteristics, as stratified by age, are seen in Table 1. Among the entire cohort, 169 (51.7%) were ≥70 years of age (“older adults”), 170 (52.0%) were female, 243 (74.3%) had adenocarcinoma as the histological subtype, and 33 (7.6%) were never smokers. Furthermore, the number of patients with poor ECOG PS (ECOG ≥ 2), brain metastases and baseline autoimmune disease at treatment initiation were 86 (26.3%), 44 (13.5%), and 75 (22.9%), respectively.

Older adults were more likely to have a lower stage of disease at the time of their initial diagnosis (*p* = 0.03), all other characteristics were not statistically significant. In particular, the ECOG status, presence of brain metastases, and underlying autoimmune conditions at treatment initiation were not statistically different between the two age cohorts (*p* = 0.84, *p* = 0.06, and *p* = 0.06, respectively).

### 3.2. Clinical Outcomes

The relevant clinical outcomes are summarized in Table 2. At the time of data cut-off, 41.0% of patients were alive, with a median follow-up time of 19.2 months, ranging from 2.7 to 41.7 months. For the entire cohort, the median OS (mOS) was 11.2 months (95% CI 8.8–15.3), median TTF (mTTF) was 3.1 months (95% CI 3.1–4.9), and ORR was 32.0%.

**Figure 1:**
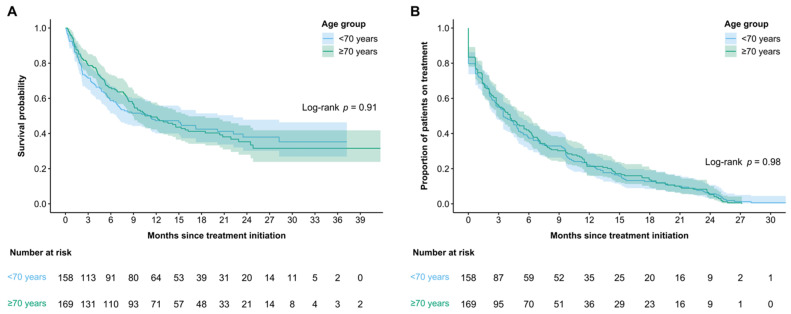
Kaplan−Meier curves for median: (**A**) overall survival (OS) and (**B**) time to treatment failure (TTF) by age.

When comparing the outcomes of the older and younger adults, we did not find difference in mOS, TTF, or ORR. The mOS was 11.3 months (95% CI 8.9–16.2) in older adults versus 11.2 (95% CI 6.7–22.2) months in younger adults (*p* = 0.91) (Figure 1A). The TTF was 4.1 months (95% CI 2.8–6.0) in older adults versus 3.46 (95% CI 2.7–5.0) months in younger adults (*p* = 0.98) (Figure 1B). The ORR was 30.8% in older adults versus 33.3% in younger adults (*p* = 0.67).

An exploratory analysis of the landmark survival times did not demonstrate differences in the 3-, 12-, or 24-month cut-offs (*p* = 0.21; *p* = 0.78; *p* = 0.95, respectively). However, for the entire cohort, we observed 3-, 12-, and 24-month survival rates of 74.6%, 41.3%, and 12.5%, respectively.

After the cessation of pembrolizumab, the rates of subsequent therapy were significantly higher in the younger adult cohort (24.1%) compared with the older adult cohort (13.7%) (*p* = 0.02).

### 3.3. Safety Data

Safety data are found in Table 3. In the overall cohort, the rate of any significant irAE was 26.6%, with the three most common being pneumonitis (32.2% of all irAE), colitis (14.9% of all irAE), and arthritis (13.8% or irAE). A total of 24 irAE directly led to hospitalization.

There were no differences in the rates of irAE between younger adults (26.6%) and older adults (26.6%) (*p* = 0.99), nor were there differences in irAE-related hospitalizations between younger adults (30.2%) and older adults (25.6%) (*p* = 0.63)

Finally, there were no differences in the rates of all-cause hospitalization between younger and older adults. The rates of hospitalizations from any cause were 44.9% in younger adults versus 46.7% in older adults (*p* = 0.74).

Notably, in a sensitivity analysis with 75 years as the cut-off age, the clinical outcomes and safety outcomes were similar to our primary analysis, with no statistical difference between the two age groups (Supplemental Tables S1 and S2).

### 3.4. Prognostic Factors of Overall Survival

On the univariable analyses, we found that ECOG ≥ 2, the presence of liver metastases, BMI < 30, an elevated platelet−leukocyte ratio, intermediate/poor lung immune prognostic index (LIPI), low albumin, and low hemoglobin had a signal of association (defined as *p* < 0.2) with a worse OS (Supplemental Table S3). Notably, age was not a prognostic factor (*p* = 0.91). As such, these factors were included in the multivariable model (Table 4). After adjusting for other relevant prognostic factors, ECOG ≥ 2 (HR 2.49; 95% CI 1.74–3.57, *p* < 0.001), BMI < 30 (HR 1.63; 95% CI 1.01–2.60 *p* = 0.04), intermediate/poor LIPI (HR 1.84; 95% CI 1.26–2.68, *p* = 0.001), hypoalbuminemia (HR 1.48; 95% CI 1.03–2.14, *p* = 0.04), and anemia (HR 1.45; 95% CI 1.01–2.07, *p* = 0.04) were still significantly associated with worse OS.

## 4. Discussion

Data from KEYNOTE-024 have solidified single-agent pembrolizumab as a standard of care for patients with PD-L1 high NSCLC. However, it is well known that patients treated in the real world differ from clinical trial patients in their underlying demographics and clinical status, including being of an older age. Older age is a particularly relevant demographic, given the high incidence and prevalence of lung cancer in older adults [15]. In NSCLC, several previous studies have evaluated the impact of age on the safety and efficacy of immune checkpoint inhibitors. For example, Galli et al. (2019) [10], Youn et al. (2020) [11], and Marur et al. (2018) [12] all previously demonstrated a comparable safety and efficacy of immunotherapy between younger and older cohorts. While these studies had large cohorts, they lacked therapeutic consistency by including multiple immunotherapeutic agents and different lines of treatment (i.e., first versus second line). In our current study, we selectively examined the impact of age on patients receiving pembrolizumab as a first-line therapy. To our knowledge, this is the largest study examining the impact of age in this particular cohort.

Overall, older adults (≥70 years of age) and younger adults had an equivalent ORR, TTF, and mOS. Notably, younger adults were statistically more likely to receive subsequent treatment. It is unclear why this happened; one possibility is that older adults may choose to pursue palliation after treatment failure while younger adults may choose to attempt additional therapy. Another possibility is that older adults tend to have more comorbidities, which could lead to hesitancy in patients and clinicians for trying additional harsh therapies. With regards to safety, the irAE rates were equivalent between the younger and older cohorts. Hospitalizations, both directly due to an irAE and all-cause hospitalizations, were equal in both cohorts. Together, the effectiveness and safety of pembrolizumab were equivalent between older and younger adults. These results are consistent with previous studies [10,11,12].

Despite the equivalent effectiveness and safety profiles between the two cohorts, the short-term mortality in both groups was surprisingly high. At 3-months after treatment initiation, the overall mortality rate was 25% and was similar in both age cohorts. At 12-months, the overall mortality rate was 60%. This is much higher than the mortality rates quoted in the landmark KEYNOTE-024 trial [3]. Additionally, mOS and ORR were lower in our cohort (11 months and 32%, respectively) compared with the KEYNOTE-024 cohort (26 months and 45%, respectively) [4]. A possible explanation for the increased early mortality, shorter overall survival, and poorer ORR is the discrepancy in the baseline health of real-world versus clinical trial cohorts. Our group previously investigated this phenomenon and found that 32% of “real-world” patients treated with immunotherapy for renal-cell carcinoma, NSCLC, or melanoma would not be eligible for corresponding immunotherapy trials due clinical and laboratory exclusion criteria, including ECOG. The patients who would be ineligible had a lower median overall survival and ORR compared with the eligible patients [16]. In the current study, there was a high proportion of poor ECOG status patients (>25%), suggesting a possible explanation for the increased short-term mortality. This is consistent with previous research that shows many patients in real-world settings have an ECOG-PS ≥ 2, and that these patients have worse outcomes on immunotherapy [17]. Additionally, the subjective assessment of the performance status is challenging and, as a result, there may be an underestimation of poor PS. Another possible contributing factor in the discrepancy between our cohort and the KEYNOTE-024 cohort is the proportion of non-smokers (7.6% versus 3%) [3]. Retrospective analyses have demonstrated that non-smokers are less likely to benefit from first-line pembrolizumab monotherapy compared with former or current smokers [18,19,20]. It has been suggested that a higher tumor mutational burden generated by cigarette carcinogens creates a more immunogenic milieu, thereby providing improved rates of immunotherapy response. The difference in survival and response rates between real-world and clinical trial populations should be further investigated, as it could be helpful to identify patients at high risk of shorter survival times, so that patients can be spared the potential toxicities of a futile therapy, and supportive care can be implemented.

A key secondary objective of our study was to characterize the baseline clinical/pathologic factors associated with survival outcomes. Our multivariable analysis indicated that ECOG ≥ 2, BMI < 30, intermediate/poor LIPI, hypoalbuminemia, and anemia were all associated with worse survival outcomes. An ECOG status of ≥2 has previously been shown to be associated with decreased median survival with immunotherapy [17]. Based on our current study, ECOG ≥ 2 is the strongest predictor of reduced survival. Notably, patients with a poor ECOG status were excluded from the KEYNOTE-024 trial [3], which again could explain the high short-term mortality in our patient population. Another strong predictor of survival was a poor LIPI score; LIPI is a previously defined prognostication metric that utilizes the derived neutrophil-to-lymphocyte ratio (dNLR) and lactate dehydrogenase (LDH). Our results are consistent with previous research showing that a poor or intermediate baseline LIPI correlates with worse outcomes for immunotherapy [21,22]. A BMI of <30 was also associated with decreased survival in our study, supporting the well-established obesity paradox. Obesity has previously been found to be protective in cancers associated with wasting such as lung cancer, renal cell carcinoma, and melanoma—interestingly, cancers predominantly treated with immunotherapy, potentially due to the increased efficacy of the PD-1/PD-L1 blockade [23,24,25].

Overall, an age of <70 versus ≥70 years was not a prognostic factor in lung cancer patients treated with pembrolizumab. Rather, we found several clinical and laboratory features described above that impacted survival, independent of age. Furthermore, the effectiveness and safety as measured by mOS, mTTF, ORR, and irAEs were also comparable between older and younger patients, indicating that age should not be a barrier for these patients accessing treatment with pembrolizumab. Future studies should investigate why both short-term mortality and median OS were poorer in our cohort compared with the corresponding clinical trials. Developing the capacity to predict who will benefit from immunotherapy based on the aforementioned prognostic features could prove extremely clinically useful.

As with all retrospective studies, a limitation in our study is missing information not included in physician charting. Furthermore, we only collected data on the most significant irAE, based on the need for steroid therapy or hospitalization. Additionally, we treated age as a dichotomous variable for the purpose of our analysis, while in reality it is continuous. However, this is standard practice among similar studies [8,12].

## 5. Conclusions

In conclusion, age does not impact the effectiveness or safety of pembrolizumab in NSCLC, and therefore advanced age should not be a deterrent to treating these patients with pembrolizumab. Physicians and care providers can thus focus on other factors that may influence therapeutic outcomes, aside from age. For example, ECOG ≥ 2, BMI < 30, intermediate/poor LIPI, hypoalbuminemia, and anemia were all associated with worse survival outcomes. Finally, although we elucidated the factors associated with improved survival, the short-term survival in our population was much lower than expected based on the clinical trials and this is an area that should be investigated further.

## Figures and Tables

**Table 1:** Distribution of the baseline clinical and pathological characteristics by age.

Characteristic	Age		*p*-Value
	<70 (*n* = 158)	≥70 (*n* = 169)	
Sex–no. (%)			0.19
Female	88 (55.7)	82 (48.5)	
Male	70 (44.3)	87 (51.5)	
Smoking status—no. (%)			0.87
Never	12 (7.9)	12 (7.4)	
Ever	140 (92.1)	150 (92.6)	
Histology—no. (%)			0.06
Adenocarcinoma	125 (79.6)	118 (69.8)	
Squamous	22 (14.0)	41 (24.3)	
Other	10 (6.4)	10 (5.9)	
Stage at Diagnosis—no. (%)			0.03
I/II	7 (4.4)	21 (12.4)	
III	43 (27.2)	46 (27.2)	
IV	108 (68.4)	102 (60.4)	
KRAS Status—no. (%)			0.10
Wildtype	23 (14.6)	14 (8.3)	
Mutated	23 (14.6)	19 (11.2)	
Unknown	112 (70.9)	136 (80.5)	
Autoimmune Disease—no. (%)			0.06
No	128 (81.5)	122 (72.6)	
Yes	29 (18.5)	46 (27.4)	
ECOG PS—no. (%)			0.84
<2	116 (73.4)	125 (74.4)	
≥2	42 (26.6)	43 (25.6)	
Brain Metastases—no. (%)			0.06
No	129 (82.7)	152 (89.9)	
Yes	27 (17.3)	17 (10.1)	
Liver Metastases—no. (%)			0.23
No	127 (80.9)	145 (85.8)	
Yes	30 (19.1)	24 (14.2)	
LIPI—no. (%)			0.03
Good	39 (32.8%)	60 (46.2%)	
Poor/intermediate	80 (67.2%)	70 (53.8%)	

Abbreviations: KRAS = Kirsten rat sarcoma viral oncogene; ECOG PS = Eastern Oncology Group Performance Status; LIPI = Lung Immune Prognostic Index.

**Table 2:** Summary data of the relevant clinical outcomes based on age (cut-off 70 years-old).

Clinical Outcome	Overall	Age		*p*-Value
		<70 (*n* = 158)	≥70 (*n* = 169)	
ORR—no. (%)	79 (32.0)	38 (33.3)	41 (30.8)	0.67
mTTF—months (95% CI)	3.91 (3.06–4.86)	3.46 (2.73–4.99)	4.14 (2.76–5.98)	0.98
mOS—months (95% CI)	11.24 (8.77–15.31)	11.2 (6.74–22.2)	11.3 (8.87–16.2)	0.91
Landmark Analyses—no. (%)				
3 month	244 (74.6)	113 (71.5)	131 (77.5)	0.21
12 month	135 (41.3)	64 (40.5)	71 (42.0)	0.78
24 month	41 (12.5)	20 (12.7)	21 (12.4)	0.95
Subsequent Treatment—no. (%)	61 (18.7)	38 (24.1)	23 (13.7)	0.02

Abbreviations: ORR = overall response rate; mTTF = median time to treatment failure; mOS = median overall survival.

**Table 3:** Summary data of the safety outcomes based on age.

Safety Outcomes	Age		*p*-Value
	<70 (*n* = 158)	≥70 (*n* = 169)	
Any Significant IrAE—no. (%)	42 (26.6)	45 (26.6)	0.99
Significant IrAE—no. (%)			0.60
Pneumonitis	12 (28.6)	16 (35.6)	
Colitis	8 (19.0)	5 (11.1)	
Arthritis	5 (11.9)	7 (15.6)	
Dermatologic	6 (14.3)	3 (6.7)	
Hepatitis	1 (2.4)	3 (6.7)	
Thyroiditis	1 (2.4)	2 (4.4)	
Adrenal insufficiency	0 (0.0)	2 (4.4)	
Other	9 (21.4)	7 (15.6)	
irAE Hospitalization—no. (%)	13 (30.2)	11 (25.6)	0.63
Any Hospitalization—no. (%)	71 (44.9)	79 (46.7)	0.74

Abbreviations: irAE = immune related adverse events.

**Table 4:** Multivariate analysis of the clinical and laboratory factors associated with OS.

Characteristic	OS HR (95% CI)	*p*-Value
Age		
<70 (reference) versus ≥70	0.89 (0.62–1.26)	0.50
ECOG		
<2 (reference) versus ≥2	2.49 (1.74–3.57)	<0.001
Liver Metastases		
No (reference) versus Yes	1.06 (0.69–1.62)	0.79
BMI		
<30 (reference) versus ≥30	1.63 (1.01–2.60)	0.04
PLR		
<180 (reference) versus ≥180	0.85 (0.56–1.30)	0.45
LIPI		
Good (reference) versus Int./Poor	1.84 (1.26–2.68)	0.001
Albumin		
≥33 (reference) versus <33	1.48 (1.03–2.14)	0.04
Hemoglobin		
≥130 (reference) versus <130	1.45 (1.01–2.07)	0.04

## Data Availability

Data will not be shared, as the ethics approval for this project does not allow for sharing of this data due to patient privacy and confidentiality.

## Supplementary Materials

The following are available online at https://www.mdpi.com/article/10.3390/curroncol28050357/s1. Table S1: Sensitivity analysis summary data of relevant clinical outcomes based on age (cut-off 75 years-old). Table S2: Sensitivity analysis summary data of safety outcomes based on age (cut-off 75 years-old). Table S3: Univariate analysis of clinical and laboratory factors associated with OS.
