# Supplementary material for: Effectiveness and Safety of First-Line Pembrolizumab in Older Adults with PD-L1 Positive Non-Small Cell Lung Cancer: A Retrospective Cohort Study of the Alberta Immunotherapy Database
Source: Curr Oncol. 2021 Oct 18;28(5):4213–22. doi: 10.3390/curroncol28050357 (PMC8534423; doi:10.3390/curroncol28050357)
Supplement: Supplementary file 1 [file curroncol-28-00357-s001.zip › curroncol-1401920-supplementary.pdf]

Supplementary Materials

Table S1. Sensitivity analysis summary data of relevant clinical outcomes based on age (cut-off 75 years-old).

Clinical Outcome	Overall	Age		*p*-Value
		<75 (*n* = 248)	≥75 (*n* = 79)	
**ORR – no. (%)**	79 (32.0)	58 (31.4)	21 (33.9)	0.71
**mTTF – mo. (95% CI)**	3.91 (3.06–4.86)	3.58 (2.76–4.80)	5.52 (2.76–7.16)	0.90
**mOS – mo. (95% CI)**	11.24 (8.77–15.31)	11.3 (7.79–16.2)	10.5 (8.28–20.8)	0.99
**Landmark Analyses – no. (%)**				
3 mo	244 (74.6)	179 (72.2)	65 (82.3)	0.07
12 mo	135 (41.3)	103 (41.5)	32 (40.5)	0.87
24 mo	41 (12.5)	34 (32.7)	7 (8.9)	0.26
**Subsequent Treatment – no. (%)**	61 (18.7)	53 (21.4)	8 (10.3)	0.03

Table S2. Sensitivity analysis summary data of safety outcomes based on age (cut-off 75 years-old).

Safety Outcomes	Age		*p*-Value
	<70 (*n* = 248)	≥70 (*n* = 79)	
Any Significant IrAE – no. (%)	60 (24.2)	27 (34.2%)	0.08
Significant IrAE – no. (%)			0.37
Pneumonitis	19 (31.7)	9 (33.3%)	
Colitis	10 (16.7)	3 (11.1%)	
Arthritis	9 (15.0)	3 (11.1%)	
Dermatologic	7 (11.7%)	2 (7.4%)	
Hepatitis	2 (3.3%)	2 (7.4%)	
Thyroiditis	1 (1.7%)	2 (7.4%)	
Adrenal insufficiency	0 (0.0%)	2 (7.4%)	
Other	12 (20.0%)	4 (14.8%)	
irAE Hospitalization – no. (%)	19 (31.7%)	5 (19.2%)	0.24
Any Hospitalization – no. (%)	109 (44.0%)	41 (51.9%)	0.22

Table S3. Univariate analysis of clinical and laboratory factors associated with OS.

Characteristic	OS HR (95% CI)	*p*-Value
**Age**		
<70 (reference) vs. ≥70	0.98 (0.74–1.30)	0.91
Autoimmune Disease		
No (reference) vs. Yes	1.00 (0.72–1.40)	0.98
ECOG		
<2 (reference) vs. ≥2	2.85 (2.11–3.83)	<0.001
Brain Metastases		
No (reference) vs. Yes	1.09 (0.72–1.64)	0.69
Liver Metastases		
No (reference) vs. Yes	1.61 (1.14–2.28)	0.008
BMI		
<30 (reference) vs. ≥30	1.69 (1.16–2.47)	0.006
PLR		
<180 (reference) vs. ≥180	1.49 (1.05–2.13)	0.03
LIPI		
Good (reference) vs. Int./Poor	2.53 (1.80–3.55)	<0.001
**Albumin**		
≥33 (reference) vs. <33	1.90 (1.36–2.64)	<0.001
**Hemoglobin**		
≥130 (reference) vs. <130	1.84 (1.35–2.49)	<0.001
